# A Mechanical Anæsthetic

**Published:** 1871-02

**Authors:** 


A Mechanical Anæsthetic.—The *Practitioner* for December contains an interesting article by the late Dr. A. Waller on the compression of the vagus nerve as a means of producing anæsthesia. It is claimed that by vagal pressure the patient can be speedily and safely brought into a state of complete insensibility, and that several surgical operations have been performed with entire absence of pain. It appears to produce a condition specially favorable to the reduction of dislocated bones. Attention was directed to this subject by Dr. Waller, shortly before his death, in the hope that it might be found of practical value in a large number of cases where ether is now required. This may be only the revival of an old fashion, as the Assyrians are reported to have produced anæsthesia by compression of the cervical vessels during the performance of circumcision.

